# The Effect of Botulinum Toxin-A on Chronic Muscle-Related Pain in Cerebral Palsy

**DOI:** 10.3389/fneur.2022.936625

**Published:** 2022-06-03

**Authors:** Christian Wong

**Affiliations:** ^1^Hvidovre Hospital, Hvidovre, Denmark; ^2^Department of Orthopedics, Hvidovre University Hospital, Hvidovre, Denmark

**Keywords:** pain, musculoskeletal pain, cerebral palsy, botulinum toxins type A, ultrasound injections, evaluation methods

## Introduction

We congratulate the authors for the insightful article “A First Clinical Trial on Botulinum Toxin-A for Chronic Muscle-Related Pain in Cerebral Palsy,” which I read with great interest (1). They performed a researcher-initiated and academically funded, randomized, placebo-controlled double-blind clinical trial. This is a strong design, which has to be applauded since there is a lack of evidence using such a study design for Botulinum boxin-A (BoNT/A) for chronic muscle-related pain. They stopped their study at an independent interim analysis, due to not having the probability of showing treatment superiority of efficacy of BoNT-A for chronic muscle-related pain.

## Discussion

However, it is important to nuance and perspective their results, since the clinical and research methods in this study potentially could have affected the outcome. We will discuss these factors:

### Injection Methods

In the study, the treatment consisted of one session of electromyographically (EMG) guided intramuscular injections of either BoNT-A or placebo. The injection method is highly important for the effect of the injected medicine since this might affect the delivery of medicine into the targeted muscle since EMG does not always ascertain that the needle is in the right muscle (2). Here, electrical stimulation is a better method of verifying the right muscle (2). However, using electric stimulation may need several needle replacements and can be painful. As an alternative ultrasound can be used to ensure the accuracy of injections (2). We utilized ultrasound for muscle identification when injecting BoNT-A for pain in children with cerebral palsy in the article “Measuring Effects on Pain and Quality of Life after Abobotulinum Toxin A Injections in Children with Cerebral Palsy” (3).

### Targeting Painful Muscle

In our study, our targeted muscles were almost similar to the ones in the current discussed study except for the main culprit for muscle pain in our study, namely the psoas muscle of the iliopsoas muscle. This was a muscle that consistently was painful. We find it interesting that there would be such a difference between children and adults with cerebral palsy. The injections would have been unfeasible without ultrasound in our study since the medicine was delivered in the psoas muscle at the level of the hip capsule, which is immensely small at that level.

In our study, we found that evaluating muscle-related pain is very difficult and especially for the psoas muscle, where the pain is identified by eliciting pain when performing Thomas' test with a swift movement and by direct palpation. In general, 60% of children with cerebral palsy experience pain within the last week, and this is (often) unidentified and untreated (4), and having pain is strongly associated with low quality of life (5). In our study, we focused on the method of evaluation for pain and the method of targeting the muscles for injection.

### Pain Evaluation Methods

This warrants appropriate pain evaluation methods to meet i.e., challenges of sometimes non-communicative patients with a handicap. The preferable methods are self-reporting and using multiple and validated “pain assessment tools” (6). In the discussed study, we applaud the authors for using appropriate methods for pain evaluation. In our study, we evaluated the localized pain injection treatment by a localized pain evaluation method by clinical examination (the modified R-FLACC). We found this prudent since there can be many “modalities of pain such as intra-articular pain and neuropathic pain” (1), and for evaluating the specific effect on pain by the specific localized treatment, local evaluation is warrented to discriminate between pain relief for this specific treatment amongst the other types of pain. In the currently discussed study, the “participants were interviewed and assessed for muscle-related pain and examined for regional spastic muscles to be targeted for injection” (1). In a personal communication with the main author, their evaluation of the primary parameter is still not clear to me. I would kindly ask them to comment on this important point.

### Interrelation of Spasticity and Pain/Mechanisms of Analgesia for BoNT-A

When evaluating muscle-related pain, our experience is that relating pain strictly to spasticity might not be prudent, since “proposed mechanisms of analgesia include altered neurotransmitter release of sensory nerves and central modulatory effects, hence not necessarily related to spasticity” (1). In the currently discussed study, they experienced that “the supposed effect (for pain?) of BoNT-A could be through other mechanisms than spasticity reduction.” They found that “pain relief does not necessarily coincide with muscle relaxation when BoNT-A is used for established pain indications” and “modulation of peripheral neurotransmitter release, anti-inflammation, and central nervous system modulatory effects have been proposed as alternative modes of action in BoNT-A–mediated pain relief” (1), hence to interrelate pain to spasticity might lead to inadequate *targeting* and subsequent evaluation of outcome in muscle-related pain.

In conclusion, this is an important article with a strong design, but for the above reasons, we find that further research is needed that takes the above factors into account—especially concerning the targeting of injected muscle, injection techniques and evaluation methods. For full disclosure, we performed a sponsored study using BoNT-A for chronic muscle-related pain in children with cerebral palsy (3).

## Author Contributions

The author confirms being the sole contributor of this work and has approved it for publication.

## Funding

The author declares that the research was conducted a sponsored study using BoNT-A for chronic muscle-related pain in children with cerebral palsy, Tracking Number: A-SE-52120-240, Study Title: Measuring effects on pain and quality of life after dysport^®^ injection in ‘almost’ toxin naive cerebral palsy children - follow-up from post-injection to 28 weeks.

## Conflict of Interest

The author declares that the research was conducted in the absence of any commercial or financial relationships that could be construed as a potential conflict of interest.

## Publisher's Note

All claims expressed in this article are solely those of the authors and do not necessarily represent those of their affiliated organizations, or those of the publisher, the editors and the reviewers. Any product that may be evaluated in this article, or claim that may be made by its manufacturer, is not guaranteed or endorsed by the publisher.
